# Cerebral perfusion and gray matter volume alterations associated with cognitive impairment in metabolic syndrome with cerebral small vessel disease

**DOI:** 10.3389/fnagi.2025.1538850

**Published:** 2025-04-22

**Authors:** Han Liu, Wenhui Zheng, Jie Geng, Xia Zhou, Yu Xia, Yan Liu, Gang Zhang, Xiaoqun Zhu, Daping Lv, Zhongwu Sun

**Affiliations:** ^1^Department of Neurology, The First Affiliated Hospital of Anhui Medical University, Hefei, China; ^2^Department of Neurology, Anhui Public Health Clinical Center, Hefei, China; ^3^Department of Neurology, Tongren Hospital, Shanghai Jiao Tong University School of Medicine, Shanghai, China; ^4^Department of General Medicine, The Second Affiliated Hospital of Bengbu Medical University, Bengbu, China; ^5^Department of Neurology, The Third People’s Hospital of Hefei, Hefei, China; ^6^Department of Neurology, Suzhou Hospital of Anhui Medical University, Suzhou, China; ^7^Department of Rehabilitation, Anhui Public Health Clinical Center, Hefei, China

**Keywords:** metabolic syndrome, cerebral perfusion, gray matter volume, cerebral small vessel disease, cognitive impairment

## Abstract

**Objective:**

Metabolic syndrome (MetS) combined with cerebral small vessel disease (CSVD) is associated with cognitive dysfunction. However, the underlying mechanisms remain poorly understood. This study investigated the roles of brain perfusion and gray matter volume (GMV) in cognitive dysfunction in patients with MetS combined with CSVD (MetS-CSVD).

**Methods:**

This study enrolled 180 MetS patients and 86 healthy controls (HCs). Patients with MetS were categorized into MetS without CSVD (MetS-NCSVD) (*n* = 58) and MetS with CSVD (MetS-CSVD) (*n* = 122) groups. All participants underwent laboratory tests, neuropsychological assessments, 3D pseudo-continuous arterial spin labeling (3D-PCASL), and magnetic resonance imaging scans with voxel-based morphometry (VBM). Cerebral blood flow (CBF), GMV, and clinical scales were analyzed for correlations in regions of interest (ROIs). The relationships among cognitive performance, CBF, and GMV were evaluated using partial correlation analysis and multivariable linear regression analysis (MLRA). Mediation analysis was performed to investigate the roles of CBF and GMV in the association between CSVD scores and cognitive performance.

**Results:**

Compared to the MetS-NCSVD and HC groups, the MetS-CSVD group displayed significantly reduced perfusion and GMV in the frontal and temporal lobes. Partial correlation analysis revealed that CBF and GMV in the frontotemporal lobe were strongly associated with overall cognitive function, executive function, and language function in the MetS-CSVD group. MLRA identified the CSVD score as the sole independent factor negatively associated with cognitive performance. Mediation analysis revealed that CBF and GMV in the frontal lobes mediated the relationship between the CSVD score and overall cognitive and executive function.

**Conclusion:**

CBF and GMV were strongly associated with cognitive performance. CSVD, rather than individual components of MetS, mediated cognitive impairment in patients with MetS-CSVD. CSVD likely contributes indirectly to cognitive impairment through its effects on CBF and GMV in these patients.

## 1 Introduction

Metabolic syndrome (MetS) is a complex condition characterized by metabolic disorders, including diabetes, hypertension, hypertriglyceridemia, dyslipidemia, and abdominal obesity (Neeland et al., 2024). Vascular risk factors, such as obesity, dyslipidemia, hypertension, and diabetes, are contributors to dementia and cognitive impairment (Breteler, 2000; Biessels et al., 2008; Solomon et al., 2009). A longitudinal population study of 2,097 Italian individuals aged 65–84 years, evaluated over 3.5 years, found that patients with MetS were more likely to progress to dementia than those without MetS. After multifactorial adjustments, patients with MetS had approximately double the risk (Solfrizzi et al., 2011). Similarly, patients with MetS have a higher risk of cognitive decline and impairment and dementia (Forti et al., 2010; Feng et al., 2013; Siervo et al., 2014). The underlying mechanisms can include endothelial and cellular dysfunction, atherosclerosis, asymptomatic ischemic brain injury, activation of thrombotic and oxidative stress, insulin resistance, chronic microinflammation, and dysregulation of the renin-angiotensin-aldosterone axis (Waldstein et al., 2004). Advances in medical technology and increased life expectancy have contributed to a rising prevalence of dementia. However, the mechanisms by which MetS contributes to cognitive impairment remain poorly understood. Our previous study identified cerebral white matter hyperintensities (WMHs) as mediators of MetS-related cognitive impairment (Zheng et al., 2023). WMHs, frequently observed in older adults, are commonly attributed to vascular disease (Garnier-Crussard et al., 2023), which is prevalent in most patients with MetS.

Cerebral small vessel disease (CSVD) involves pathological changes in small intracranial vessels, including arteries, arterioles, capillaries, and veins (Elahi et al., 2023). Under physiological conditions, these vessels play crucial roles in blood transport, cerebral blood flow regulation, maintaining the blood–brain barrier, and facilitating intercellular fluid exchange. CSVD appears as white matter hyperintensities (WMHs), lacunar infarcts (LI), cerebral microbleeds (CMBs), brain atrophy, and enlarged perivascular spaces (EPVS) on magnetic resonance imaging (MRI) (Jiang et al., 2022). Components of MetS, including hypertension, elevated blood glucose levels, and dyslipidemia, can adversely affect the cerebral vascular system, contributing to CSVD (Kalaria, 2016). MetS is linked to the incidence and severity of cerebrovascular disease, particularly subcortical microvascular disease, which is related to cognitive decline (Viscogliosi et al., 2016). MetS has also been linked to asymptomatic cerebral infarctions and white matter lesions (Bokura et al., 2008), doubling the morbidity of such lesions (Park et al., 2007). Diabetes is associated with both LI and WMH (Liu et al., 2018; Wang et al., 2020). Diabetes and hypertension are linked to periventricular WMH (Yin et al., 2014). MetS and its components, including hyperlipidemia, hypertension, and diabetes, are risk factors for EPVS (Wu et al., 2020). In summary, MetS-related cognitive impairment may involve cerebrovascular lesions. Therefore, exploring the mechanisms of cognitive impairment in patients with MetS with CSVD (MetS-CSVD) is crucial for early prevention, diagnosis, and improving prognosis and quality of life.

In a healthy brain, approximately one-third of blood oxygen is delivered to the brain parenchyma via capillaries, small arteries, and arterioles, supporting brain metabolism. Cerebral blood flow (CBF) microcirculation delivers essential nutrients to brain tissue while removing metabolic waste. Therefore, CBF may be a valuable neuroimaging biomarker, as hemodynamic alterations can highlight vulnerable brain areas that are not visible on structural imaging techniques. Patients with CSVD exhibit abnormal CBF patterns (Neumann et al., 2022). However, it remains unknown whether CBF mediates the regulation of CSVD in relation to cognitive performance in patients with MetS-CSVD. Currently, arterial spin labeling (ASL) is a commonly used non-invasive approach for CBF measurement (Delvecchio et al., 2022). Methods for studying cerebral structure morphometry, including visual, semiautomatic, and manual measurements, have been highly biased, poorly reproducible, and insensitive to microstructural changes. Voxel-based morphometry (VBM) is fully automated, standardized, sensitive, accurate, and reliable (Ashburner and Friston, 2000). VBM can detect subtle structural changes in the brain that are involved in neurological dysfunction across various diseases and may help elucidate cognitive impairment mechanisms in patients with MetS-CSVD. A cross-sectional comparative study using VBM, comprising 104 patients with MetS and 104 healthy controls, found a noticeable correlation between MetS and reduced gray matter volume (GMV) in multiple discrete brain regions (Kotkowski et al., 2019). No studies have investigated the relationship between cerebral structure and cognitive performance in patients with MetS-CSVD using neuroimaging methods. Based on previous studies, we hypothesize that MetS promotes CSVD by affecting cerebral blood flow (CBF), further affecting brain structure and cognitive function. In this study, we applied ASL and VBM techniques to explore the roles and mechanisms of cerebral perfusion and brain structural changes in cognitive impairment in MetS-CSVD patients. The results suggest that decreased CBF can be improved by preventing MetS, reducing CSVD, and further improving brain structure and cognitive function. The findings reveal a novel mechanism of cognitive impairment in patients with MetS-CSVD and open new avenues for their treatment.

## 2 Materials and methods

### 2.1 Participants

In total, 180 MetS patients and 86 healthy controls (HCs) were recruited from the First Affiliated Hospital of Anhui Medical University in Hefei, China. Among the 180 MetS patients, 122 had MetS combined with CSVD (MetS-CSVD) and 58 had MetS without CSVD (MetS-NCSVD). The inclusion criteria for MetS were based on previously established methods (Alberti et al., 2005). The inclusion criteria for CSVD were as follows: (1) age 50–80 years; (2) presence of one or more characteristic clinical manifestations of CSVD, such as cognitive dysfunction, motor dysfunction, voiding disorders, sleep disorders, and so on; and (3) cranial imaging showing one or more of the characteristic imaging features of CSVD (Wardlaw et al., 2013): a. WMH grades were scored according to the most widely used Fazekas criteria. Images of deep WMH with a Fazekas score ≥2 or periventricular WMH with a Fazekas score of 3 were included; b. LI ≥ 1, defined as a round or ovoid high signal lesion of 3–15 mm on T2-weighted imaging and T2-weighted imaging with a high-signal edge in the fluid-attenuated inversion recovery (FLAIR) sequence; c. CMB ≥ 1 is characterized as a low signal lesion with 2–10 mm in diameter on magnetic susceptibility-weighted imaging. The original CSVD scoring system allocates up to 3 points, assigning 1 point for each of the LI, WMH, and CMB present, with the total score representing the cumulative burden of CSVD. To improve the evaluation of CSVD severity, we adopted a modified CSVD scoring system with the following criteria (Amin Al Olama et al., 2020): WMH was scored from 0 to 3 based on the Fazekas score; LI was scored from 0 to 3 according to the number of luminal peduncles (0 = none, 1 = 1–2, 2 = 3–5, and 3 ≥ 5); and CMB continued to be scored as 1 for presence or 0 for absence. Thus, the total possible score for the modified CSVD was 0 to 7. The inclusion criteria for HCs were as follows: (1) age 50–80 years; (2) matched by age, sex, and education; and (3) no diagnosis of MetS or CSVD. The exclusion criteria for CSVD were as follows: (1) cerebral hemorrhage; (2) history of traumatic brain injury, brain tumors, or space-occupying lesions; (3) cerebral infarcts ≥ 20 mm in diameter on MRI (Adams et al., 1993; Arsava et al., 2010); (4) non-CSVD-associated cerebral white matter hyperintensities; (5) neurodegenerative diseases; (6) mental disorders, alcohol use disorder, or drug abuse; and (7) contraindications to MRI.

### 2.2 Demographic and clinical characteristics

Data acquired from all participants included age, sex, education, occupation, personal history, medical history, medication use, and family history. Blood samples collected after participants had fasted for a minimum of 12 h were sent to the laboratory for analysis of high-density lipoprotein cholesterol (HDL-C), triglyceride (TG), and fasting blood glucose (FBG). Systolic blood pressure (SBP) and diastolic blood pressure (DBP) were measured, with a second reading taken after 5 min. These measures were taken at least twice, and the average value was used for analyses. Waist circumstance (WC) was measured by placing a tape measure around the umbilicus while participants stood upright, and the value was recorded.

### 2.3 Neuropsychological assessment

Neuropsychological assessments were performed by professional neurologists within 1 week after the MRI scan. Overall cognitive function, executive function, information processing speed, attention, language function, memory function, visuospatial function, and emotional state were assessed. The specific assessment scales were as follows: Montreal Cognitive Assessment (MoCA) for overall cognitive function; Stroop Color and Word Test (SCWT-C), Trail Making Test (TMT-B), and Verbal Fluency Test (VFT) for executive function; Auditory Verbal Learning Test (AVLT) was used to assess memory; Information processing speed was evaluated with TMT-A and SCWT; Digit Span Test (DST) for attention; Clock Drawing Test (CDT) for visuospatial ability; and Geriatric Depression Scale (GDS) for Mood.

### 2.4 MRI data acquisition

MRI scans were acquired using a 3.0-Tesla MRI system (Discovery MR750w, General Electric, Milwaukee, WI, USA) equipped with a 24-channel head coil. The primary scanning sequences included 3D-T1, T2 FLAIR, and 3D-PCASL. The specific scanning parameters for 3D-T1 were as follows: repetition time (TR) = 8.5 ms, echo time (TE) = 3.2 ms, field of view (FOV) = 256 mm × 256 mm, flip angle = 12°, and matrix size (Matrix) = 256 × 256. The scan included 188 layers with a thickness of 1 mm, and the acquisition time was 296 s. T2 FLAIR images were acquired using the following parameters: TE = 119.84 ms, TR = 9,000 ms, flip angle = 90°, Matrix = 512 × 512, FOV = 225 mm × 225 mm, and 19 consecutive axial slices with a thickness of 7 mm. The scanning parameters of SWI were: TR = 45.40 ms, TE = 23.54 ms, FOV = 240.64 × 240.64 mm, Flip Angle = 20°, and Matrix = 512 × 512. The scan included 138 layers with a thickness of 1 mm. The specific scanning parameters of ASL were: TR = 5,070 ms, TE = 11.48 ms, Matrix = 128 × 128, FOV = 240 mm × 240 mm, Flip Angle = 111°, Post Labeling Delay Time (PLD) = 2000 ms, 50 layers, 3-mm layer thickness, and an acquisition time of 294 s.

### 2.5 ASL processing analysis and calculations

The computational steps for generating CBF images were performed on the GEDE workstation, as previously described (Xu et al., 2010). ASL processing utilized magnetization-labeled hydrogen protons as endogenous tracers. The image captured after blood labeled with the tracer entered the brain tissue while the image captured without magnetization labeling was used as the control image. The perfusion difference image of a pseudo-continuous ASL (pc-ASL) was obtained by subtracting the control image from the labeled one. The three perfusion difference images were averaged and combined with the proton density-weighted control image to produce the final CBF image. Image preprocessing and CBF analysis were performed on a MATLAB-based platform using the SPM8 program.^1^ The diffeomorphic anatomical registration through the exponentiated Lie algebra (DARTEL) algorithm was used to align with the Montreal Neurological Institute spatial Positron Emission Computed Tomography (PET) image templates for spatial normalization. Gaussian smoothing kernel with a Full-Width at Half-Maximum (FWHM) of 8 mm was performed. Multiple comparisons were corrected using the cluster-level false discovery rate (FDR) method, resulting in a cluster-defining threshold of *p* < 0.001 and a corrected cluster significance of *p* < 0.05.

### 2.6 Analysis and calculations of VBM

Image preprocessing and VBM analysis were conducted utilizing the MATLAB-based platform of the SPM8 program, which can be accessed at http://www.fil.ion.ucl.ac.uk/spm. The steps were as follows: (1) image quality check and data format conversion; (2) tissue segmentation and spatial standardization: the whole-brain 3D-T1 image was segmented into white matter, gray matter, and cerebrospinal fluid and spatially standardized by alignment with the PET image template in MNI space via the DARTEL algorithm; and (3) smoothing: Gaussian smoothing kernel with an FWHM of 8 mm. The total intracranial volume (TIV) was determined by summing the volumes of white matter, gray matter, and cerebrospinal fluid. Multiple comparisons were corrected using the cluster-level false discovery rate (FDR) method, resulting in a cluster-defining threshold of *p* < 0.001 and a corrected cluster significance of *p* < 0.05.

### 2.7 Mediation analysis

To investigate the potentially mediating effects of CBF and GMV on mediating the relationship between CSVD scores and cognitive performance, a mediation analysis using the PROCESS macro was conducted^2^ (Lee et al., 2021; detail methods are available in Supporting Information online).

### 2.8 Statistical analysis

SPSS Statistics version 25.0 for Windows (IBM Corp., Armonk, USA) was utilized for all statistical analyses. The normality of the data was assessed using the Shapiro–Wilk test. Data following a normal distribution are expressed as mean ± standard deviation (x̄ ± s). Differences between the two groups were analyzed using independent samples t-tests. One-way analysis of variance (ANOVA) was used to analyze differences between the three groups, followed by the Bonferroni *post-hoc* test. For non-normally distributed, continuous variables, a rank sum test was used, and the results are expressed as medians and quartiles [M (P25, P75)]. The Mann–Whitney U-test was used to compare differences between the two groups, and the Kruskal–Wallis H-test was used to compare differences among the three groups. Categorical variables are expressed as frequencies (%) and were analyzed using the Chi-square test. The relationships between the clinical cognition score and CBF and VBM values in brain regions with significant differences were evaluated using partial correlation analysis with adjustment for age, sex, and education. MLRA was used to examine the relationship between significantly different CBF and VBM values (independent variables) and cognitive function (dependent variables). MLRA was performed using the following three models: (1) Model 1 adjusted for age, sex, and education; (2) Model 2 adjusted for the factors of the MetS based on Model 1; and (3) Model 3 adjusted for CSVD scores based on Model 2. All analyzed *p*-values were corrected for FDR, and differences were considered statistically significant with an adjusted p-value <0.05.

## 3 Results

### 3.1 Demographic, clinical data, and neuroimaging characteristics

We recruited 180 patients with MetS [including MetS-CSVD (*n* = 122) and MetS-NCSVD (*n* = 58)] and 86 control participants. The demographic and clinical data, as well as the neuroimaging characteristics of the participants included in this study, are summarized in Table 1. Compared to the HC group, the MetS group had higher rates of hypertension, diabetes, and hyperlipidemia, significantly larger WC, TG, SBP, DBP, and FBG, and significantly lower levels of HDL-C, as well as higher CSVD scores in the MetS group. No notable differences were found among the three groups in terms of age, sex, or education. Compared to the HC group, the MetS-CSVD group had a higher percentage of hypertension, diabetes, and hyperlipidemia, significantly higher levels of WC, TG, SBP, DBP, and FBG, and significantly lower levels of HDL-C, as well as a higher CSVD score. Compared to the HC group, the MetS-NCSVD group had a higher proportion of hypertension, diabetes, and hyperlipidemia and significantly higher WC, TG, and FBG levels, while HDL-C levels were significantly lower. Compared to the MetS-NCSVD group, the MetS-CSVD group had significantly increased WC, SBP, DBP, and higher CSVD scores. The difference in total intracranial volume among the three groups was not statistically significant.

**Table 1:** Comparison of demographic, clinical data, and neuroimaging characteristics of MetS and subgroups with HC groups.

Groups	HC (*n* = 86)	MetS (*n* = 180)	MetS-NCSVD (*n* = 58)	MetS-CSVD (*n* = 122)	Inspection value	*p*-value
Demographics						
Age (y)	62.94 ± 8.66	63.89 ± 7.02^c^	62.47 ± 6.37	64.57 ± 7.24	1.992	0.138^a^
Gender (M/F)	47/39	93/87^b^	30/28	63/59	0.208	0.901^b^
Education (y)	8.58 ± 3.79	8.06 ± 4.40^c^	8.57 ± 4.01	7.84 ± 4.57	2.392	0.127^a^
Vascular risk factors						
Smoking, n(%)	17 (19.8)	48 (26.7)^b^	11(19.0)	37(30.3)	4.249	0.120^b^
Drinking, n(%)	25 (29.1)	49 (27.2)^b^	14 (24.1)	35 (28.7)	0.504	0.777^b^
Hypertension, n(%)	11 (12.8)	98 (54.4)^*,^^b^	17 (29.3)^∗^	81 (66.4)^*^^,^^&^	64.099	<0.001^b^
Diabetes, n (%)	7 (8.1)	33 (18.3)^*,^^b^	8 (13.8)^∗^	35 (28.7)^*^^,^^&^	15.171	0.001^b^
Hyperlipidemia, n(%)	6(7.0)	50 (27.8)^*,^^b^	10 (17.2)^∗^	40 (32.8)^*^^,^^&^	20.866	<0.001^b^
Coronary heart disease, n (%)	7 (8.1)	15 (8.3)^b^	6 (10.3)	9 (7.4)	0.459	0.795^b^
MetS-related characteristics						
WC (cm)	81.45 ± 6.88	87.52 ± 7.71^*,^^c^	84.16 ± 7.68^∗^	89.11 ± 7.23^*^^,^^&^	29.804	<0.001^a^
HDL-C (mmol/L)	1.58 ± 0.32	1.29 ± 0.38^*,^^c^	1.30 ± 0.39^∗^	1.28 ± 0.38^∗^	18.639	<0.001^a^
TG (mmol/L)	1.03 ± 0.34	1.73 ± 1.09^*,^^c^	1.66 ± 0.65^∗^	1.76 ± 1.25^∗^	16.825	<0.001^a^
SBP (mmHg)	126.84 ± 17.83	142.11 ± 22.26^*,^^c^	123.85 ± 17.48	152.61 ± 17.44^*^^,^^&^	63.524	<0.001^a^
DBP (mmHg)	79.18 ± 9.27	86.19 ± 14.23^*,^^c^	75.91 ± 11.73	92.10 ± 12.04^*^^,^^&^	45.299	<0.001^a^
FBG (mmol/L)	5.44 ± 0.94	6.08 ± 1.54^*,^^c^	6.15 ± 1.14^∗^	6.05 ± 1.70^∗^	6.404	0.002^a^
CSVD imaging markers						
Total intracranial volume	1482.27 ± 140.78	1489.84 ± 126.32^c^	1461.25 ± 130.37	1503.43 ± 122.55	2.156	0.118^a^
total CSVD load score	1 (0, 1)	2.5 (2, 3.25)^*,^^e^	1 (0, 1)	3 (2, 4)^*^^,^^&^	143.119	<0.001^d^

Data are presented as means ± SD, medians (interquartile range), or numbers (percentage). HC, healthy control; MetS, metabolic syndrome; CSVD, cerebral small vessel disease; MetS-NCSVD, MetS without CSVD; MetS-CSVD, MetS with CSVD; WC, Waist circumstance; HDL-C, high-density lipoprotein cholesterol; TG, triglyceride; SBP, systolic blood pressure; DBP, diastolic blood pressure; FBG, fasting blood glucose.

* Healthy control group versus MetS, MetS-CSVD, and MetS-NCSVD groups significantly different (*p* < 0.05).

& MetS-CSVD versus MetS-NCSVD group significantly different (*p* < 0.05); *p* represents the test value of three groups in HC, MetS-CSVD, and MetS-NCSVD.

a One-way analysis of variance (ANOVA) with a post-hoc Bonferroni test.

b Chi-square test.

c Two independent-samples t-test.

d Kruskal–Wallis H-test.

e Mann–Whitney U-test.

### 3.2 Group comparisons of neuropsychological assessment

The neuropsychological assessment results are provided in Table 2. The MetS group had markedly reduced overall cognitive scores (MoCA), information processing speed (TMT-A, SCWT-A, and SCWT-B), executive function (TMT-B and SCWT-C), attentional capacity (DST forwards and backward), memory function (AVLT recall and recognition), language function [VFT (animal, fruit, and vegetable categories)], and visuospatial function (CDT) than HC group. The MetS-CSVD group showed significantly lower overall cognitive scores, information processing speed, executive function, attentional capacity, memory, language, and visuospatial function than the MetS-NCSVD and HC groups. Vegetable category fluency in language function was notably lower in the MetS-NCSVD group than in the HC group. No significant differences in GDS scores were found among the three groups.

**Table 2:** Comparison of neuropsychological characteristics of the MetS group and its subgroups with the HC group.

	HC (*n* = 86)	MetS (*n* = 180)	MetS-NCSVD (*n* = 58)	MetS-CSVD (*n* = 122)	Inspection value	*p*-value
Overall cognitive performance						
MoCA	24.21 ± 3.55	22.17 ± 5.49^*,^^c^	24.52 ± 4.13	21.04 ± 5.72^*^^,^^&^	15.681	< 0.001^a^
Processing speed						
TMT-A	65.47 ± 26.83	81.06 ± 41.31^*,^^c^	63.68 ± 29.10	90.15 ± 43.88^*^^,^^&^	15.210	< 0.001^a^
SCWT-A (dot)	22.23 ± 7.63	25.91 ± 9.46^*,^^c^	23.27 ± 3.62	27.21 ± 11.07^*^^,^^&^	8.849	< 0.001^a^
SCWT-B (word)	25.48 ± 7.56	29.53 ± 11.05^*,^^c^	26.94 ± 9.40	30.83 ± 11.60^*^^,^^&^	7.653	0.001^a^
Executive function						
TMT-B	118.41 ± 54.86	148.99 ± 86.52^*,^^c^	121.69 ± 68.54	163.27 ± 93.45^*^^,^^&^	8.675	< 0.001^a^
SCWT-C (color word)	35.04 ± 11.84	39.36 ± 14.07^*,^^c^	34.96 ± 10.86	41.56 ± 14.99^*^^,^^&^	7.915	< 0.001^a^
Attention						
DST(forward)	13.29 ± 2.80	11.72 ± 3.77^*,^^c^	13.00 ± 3.24	11.10 ± 3.87^*^^,^^&^	12.128	< 0.001^a^
DST(backward)	7.24 ± 2.72	6.08 ± 2.67^*,^^c^	6.91 ± 2.57	5.67 ± 2.63^*^^,^^&^	9.902	< 0.001^a^
Memory function						
AVLT-immediate recall	15.79 ± 4.05	14.26 ± 4.47^*,^^c^	15.24 ± 3.84	13.79 ± 4.68^*^^,^^&^	5.925	0.003^a^
AVLT-delayed recall (5 min)	5.96 ± 1.75	4.81 ± 2.37^*,^^c^	5.67 ± 2.07	4.40 ± 2.40^*^^,^^&^	15.176	<0.001^a^
AVLT-delayed recall (20 min)-	5.58 ± 2.36	4.42 ± 2.45^*,^^c^	5.15 ± 2.43	3.16 ± 2.17^*^^,^^&^	15.220	<0.001^a^
AVLT-recognition	21.13 ± 2.42	19.71 ± 3.74^*,^^c^	20.90 ± 3.01	19.14 ± 3.93^*^^,^^&^	10.870	<0.001^a^
Language function						
VFT(animal)	18.98 ± 4.38	16.39 ± 5.23^*,^^c^	18.72 ± 3.80	15.25 ± 5.47^*^^,^^&^	18.568	<0.001^a^
VFT (fruit)	13.50 ± 4.68	11.40 ± 3.61^*,^^c^	13.17 ± 2.74	10.53 ± 3.68^*^^,^^&^	17.524	<0.001^a^
VFT (vegetable)	16.41 ± 4.67	13.21 ± 4.37^*,^^c^	14.86 ± 3.73	11.51 ± 3.78^*^^,^^&^	21.560	<0.001^a^
Visuospatial abilities						
CDT	8.90 ± 1.61	7.66 ± 2.94^*,^^c^	8.24 ± 1.91	7.36 ± 3.33^*^^,^^&^	8.872	<0.001^a^
Mood						
GDS	3(2, 5)	4 (2, 6)^e^	3(2, 6)	4 (2, 6.75)	3.013	0.222^d^

Data are presented as means ± SD. MMSE, Mini-Mental State Examination; MoCA, Montreal Cognitive Assessment; TMT, Trail Making Test; SCWT, Stroop Color and Word Test; DST, Digit Span Test; AVLT, Auditory Verbal Learning Test; VFT, Verbal Fluency Test; CDT, Clock Drawing Test; GDS, Geriatric Depression Scale.

* Healthy control group versus MetS, MetS-CSVD, and MetS-NCSVD groups significantly different (*p* < 0.05).

& MetS-CSVD versus MetS-NCSVD group significantly different (*p* < 0.05); *p* represents the test value of three groups in HC, MetS-CSVD, and MetS-NCSVD.

a One-way analysis of variance (ANOVA) with a post-hoc Bonferroni test.

b Chi-square test.

c Two independent-samples t-test.

d Kruskal–Wallis H-test.

e Mann–Whitney U-test.

### 3.3 Between-group comparisons of CBF

**Figure 1:**
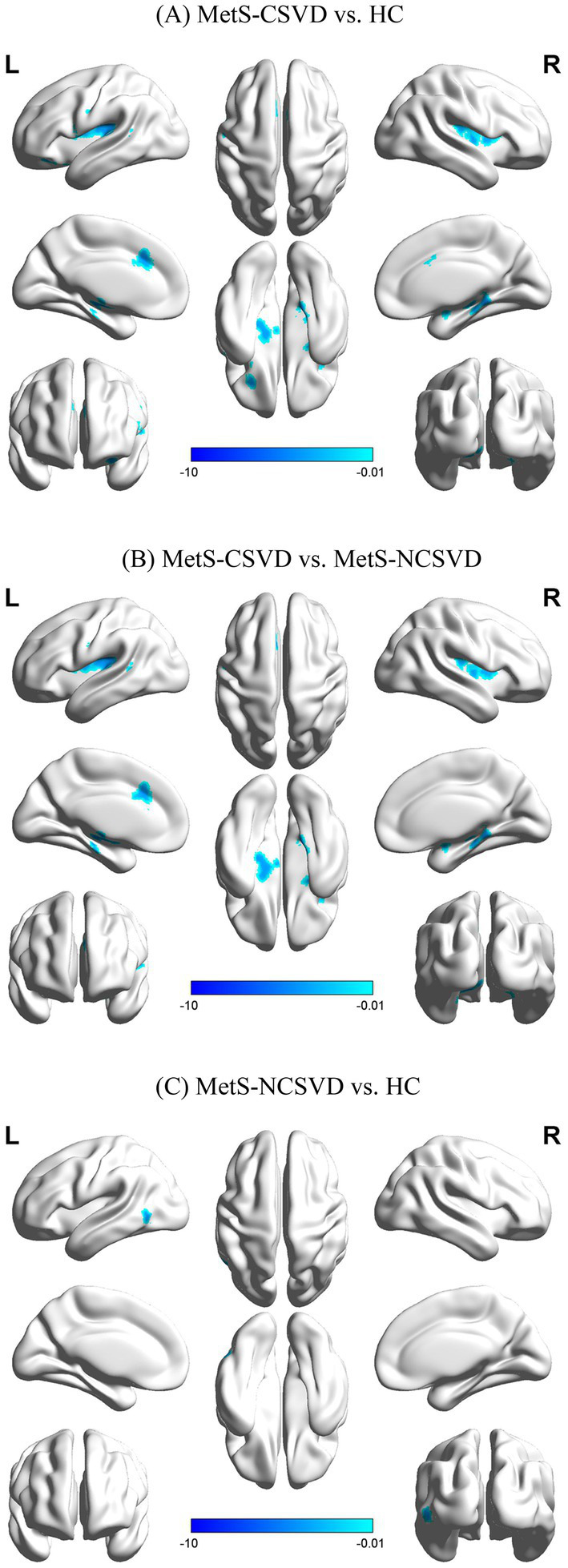
Comparison of cerebral blood flow (CBF) between groups: **(A)** MetS-CSVD vs. HC, **(B)** MetS-CSVD vs. MetS-NCSVD, and **(C)** MetS-NCSVD vs. HC. L: left brain; R: right brain; HC: healthy controls.

Age, sex, and education were used as covariates to compare CBF differences among the three groups (Table 3; Figure 1). The MetS-CSVD group showed significantly lower CBF in the MFG.R, ORBinf.L, IFGoperc.L, MTG.L, DCG.L, and ACG.L brain regions than the HC group (*p* < 0.05, FDR corrected). Contrarily, the MetS-NCSVD group showed lower CBF in the MTG.L regions (*p* < 0.05, FDR corrected). The MetS-CSVD group displayed lower CBF in MFG.R, MTG.L, DCG.L, and ACG.L regions than the MetS-NCSVD group (*p* < 0.05, FDR corrected). Although CBF reductions were observed across all three group comparisons (MetS-CSVD vs. MetS-NCSVD, MetS-CSVD vs. HC, and MetS-NCSVD vs. HC), the most pronounced difference was observed in the MetS-CSVD vs. HC group comparison.

**Table 3:** Brain regions with CBF between-group differences in MetS-CSVD, MetS-NCSVD, and HC groups.

Brain regions (AAL)	Cluster size (voxels)	Peak voxel coordinate—MNI coordinates			T-value
		x	y	z	
MetS-CSVD vs. MetS-NCSVD					
MFG.R	21	52	46	24	−3.421
MTG.L	86	−54	−64	0	−4.1691
DCG.L	141	−4	−30	38	−3.8765
ACG.L	220	−10	22	30	−4.3543
MetS-CSVD vs. HC					
MFG.R	105	34	2	58	−3.8515
ORBinf.L	108	−28	32	−14	−3.7044
IFGoperc.L	26	−46	14	6	−3.6979
MTG.L	121	−62	−52	−10	−4.0803
DCG.L	176	−4	−30	38	−4.1029
ACG.L	298	−2	26	30	−4.1219
MetS-NCSVD vs. HC					
MTG.L	77	−54	−64	0	−4.0944

AAL, Automated Anatomical Labeling; MNI, Montreal Neurological Institute; MFG.R, right middle frontal gyrus; ORBinf.L, left inferior orbitofrontal gyrus; IFGoperc.L, left opercular part of the inferior frontal gyrus; MTG.L, left middle temporal gyrus; DCG.L, left median cingulate and paracingulate gyri; ACG.L, left anterior cingulate and paracingulate gyri.

### 3.4 Groups comparison of changes in the brain structure

**Figure 2:**
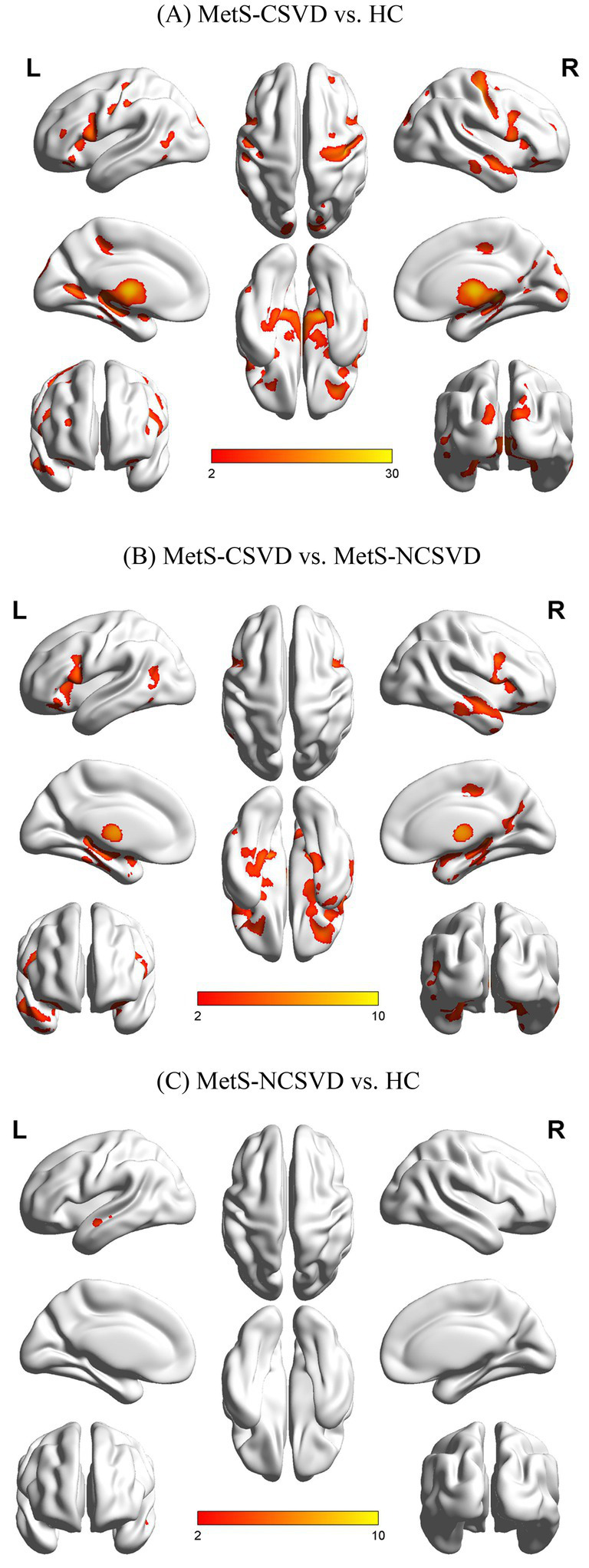
Comparison of gray matter volume (GMV) across groups: **(A)** MetS-CSVD vs. HC; **(B)** MetS-CSVD vs. MetS-NCSVD, and **(C)** MetS-NCSVD vs. HC. L: left brain; R: right brain; HC: healthy controls; FDR: false discovery rate.

Comparison of the GMV among groups, using age, sex, and education as covariates (Table 4; Figure 2), revealed that GMV was lower in the MFG.R, MTG.L, and DCG.L brain regions of the MetS-CSVD than the MetS-NCSVD group (*p* < 0.05, FDR corrected). GMV was significantly lower in the MFG.R, ORBinf.L, IFGoperc.L, MTG.L, and DCG.L regions in the MetS-CSVD group than the HC group (*p* < 0.05, FDR corrected). Contrastingly, only the MTG.L region showed lower GMV in the MetS-NCSVD group (*p* < 0.05, FDR corrected). Overall, GMV was significantly lower across the MetS-CSVD vs. HC, MetS-CSVD vs. MetS-NCSVD, and MetS-NCSVD vs. HC groups, with a pronounced decrease observed between the MetS-CSVD vs. HC group. Moreover, most of the regions with clear under perfusion similarly showed lower GMV.

**Table 4:** Brain regions with gray matter volume (GMV) differences among the MetS-CSVD, MetS-NCSVD, and HC groups.

Brain regions (AAL)	Cluster size (voxels)	Peak voxel coordinate—MNI coordinates			T-value
		x	y	z	
MetS-CSVD vs. MetS-NCSVD					
MFG.R	60	37.5	48	10.5	8.5282
MTG.L	440	−48	−57	−7.5	4.0881
DCG.L	242	−12	13.5	25.5	24.5871
MetS-CSVD vs. HC					
MFG.R	72	28.5	48	18	9.8233
ORBinf.L	1,065	−45	−21	−6	15.843
IFGoperc.L	909	−48	12	9	16.6022
MTG.L	488	−46.5	−54	−6	11.4528
DCG.L	333	−10.5	−33	45	9.4043
MetS-NCSVD vs. HC					
MTG.L	210	−52.5	−7.5	−12	3.9677

### 3.5 Multiple linear regression analysis (MLRA) of the relationship between CBF and cognitive performance

Partial correlation analysis revealed that CBF and GMV in the frontotemporal lobe were significantly related to overall cognitive function and executive function (FDR-corrected, *p* < 0.05). Cognitive impairment in the MetS-CSVD group may be modulated by alterations in CBF and GMV (Supplementary Tables 1 and 2). MLRA was subsequently performed to investigate the relationship between CBF and cognitive performance (Table 5). A strong correlation was observed between CBF and neuropsychological scores in the MetS-CSVD group (FDR-corrected, *p* < 0.05). In Model 1, MoCA was positively correlated with CBF in ORBinf.L (β = 0.611, *p* < 0.001) and MTG.L (β = 0.209, *p* = 0.004). In contrast, SCWT-C was negatively correlated with CBF in ORBinf.L (β = −0.467, *p* < 0.001), IFGoperc.L (β = −0.215, *p* = 0.009), and MTG.L (β = −0.256, *p* < 0.001), with age, sex, and education included as covariates. In Model 2, MoCA positively correlated with CBF in ORBinf.L (β = 0.643, *p* < 0.001) and MTG.L (β = 0.164, *p* = 0.041). SCWT-C negatively correlated with CBF in ORBinf.L (β = −0.453, *p* < 0.001), IFGoperc.L (β = −0.269, *p* = 0.005), and MTG.L (β = −0.226, *p* = 0.003), with age, sex, education, WC, HDL-C, TG, SBP, DBP, and FBG as covariates. Model 3 included CSVD scores and the covariates set in Model 2. The results showed that MoCA was positively correlated with CBF (β = 0.135, *p* = 0.028) and negatively correlated with CSVD scores (β = −0.814, *p* < 0.001) in ORBinf.L (Supplementary Figure 1). However, SCWT-C was positively correlated with CSVD scores (β = 0.881, *p* < 0.001) and negatively correlated with CBF (β = −0.102, *p* = 0.021) in IFGoperc.L (Supplementary Figure 1). In addition, CSVD scores showed a strong negative correlation with cognitive function (Supplementary Figure 1). CSVD scores were the only variable independently associated with neuropsychological scores, whereas none of the MetS components (SBP, DBP, WC, TG, HDL-C, and FBG) were independently associated with cognitive function.

**Table 5:** Correlation between CBF, MoCA, and SCWT-C in the MetS-CSVD group.

	Model-1					Model-2					Model-3				
	B	SE	β	t	*p*	B	SE	β	t	*p*	B	SE	β	t	*p*
MoCA															
ORBinf.L	23.492	2.721	0.611	8.632	<0.001	25.373	3.121	0.643	8.129	<0.001	5.320	2.378	0.135	2.237	0.028
MTG.L	7.749	2.630	0.209	2.946	0.004	6.120	2.946	0.164	2.078	0.041					
CSVD score											−4.293	0.318	−0.814	−13.516	<0.001
R^2^	0.504 (*F* = 56.88, *p* < 0.001)					0.501 (*F* = 43.67, *p* < 0.001)					0.827 (*F* = 213.95, *p* < 0.001)				
SCWT-C															
ORBinf.L	−40.623	7.054	−0.467	−5.759	<0.001	−41.198	8.410	−0.453	−4.899	<0.001					
IFGoperc.L	−19.897	7.524	−0.215	−2.645	0.009	−24.806	8.592	−0.269	−2.887	0.005	−9.395	3.992	−0.102	−2.354	0.021
MTG.L	−21.516	5.578	−0.256	−3.857	<0.001	−19.387	6.274	−0.226	−3.090	0.003					
CSVD score											10.732	0.527	0.881	20.383	<0.001
*R*^2^	0.564 (*F* = 50.53, *p* < 0.001)					0.568 (*F* = 40.40, *p* < 0.001)					0.893 (*F* = 377.09, *p* < 0.001)				

### 3.6 Multiple linear regression analysis (MLRA) of the relationship between GMV and cognitive performance

To further investigate the correlation between GMV and cognitive function in patients in the MetS-CSVD group, MLRA was performed while adjusting for relevant influences using three models (Table 6).The covariates used in the models were consistent with those described in Section 3.5.A significant correlation was observed between GMV and neuropsychological scores in the MetS-CSVD group (FDR-corrected, *p* < 0.05). MoCA was positively correlated with GMV in ORBinf.L (β = 0.382, *p* < 0.001 for Model 1; β = 0.316, *p* < 0.001 for Model 2; β = 0.111, *p* = 0.037 for Model 3) and IFGoperc.L (β = 0.458, *p* < 0.001 for Model 1; β = 0.523, *p* < 0.001 for Model 2; β = 0.165, *p* = 0.005 for Model 3). SCWT-C scores were negatively correlated with GMV in ORBinf.L (β = −0.389, *p* < 0.001 for Model 1; β = −0.341, *p* < 0.001 for Model 2; β = −0.112, *p* = 0.005 for Model 3) and IFGoperc.L (β = −0.447, *p* < 0.001 for Model 1; β = −0.498, *p* < 0.001 for Model 2; β = −0.097, *p* = 0.025 for Model 3). Moreover, in Model 3, MoCA scores were negatively correlated with CSVD scores (β = −0.738, *p* < 0.001), while SCWT-C scores were positively correlated with CSVD scores (β = 0.826, *p* < 0.001) (Supplementary Figure 2). Consistent with the correlation analysis of CBF and cognitive performance, CSVD scores showed a strong negative correlation with cognitive function.

**Table 6:** Correlation between gray matter volume (GMV) and MoCA and SCWT-C scores in the MetS-CSVD group.

	Model-1					Model-2					Model-3				
	B	SE	β	t	*p*	B	SE	β	t	*p*	B	SE	β	t	*p*
MoCA															
ORBinf.L	47.088	9.150	0.382	5.146	<0.001	41.485	10.954	0.316	3.787	<0.001	14.586	6.879	0.111	2.120	0.037
IFGoperc.L	44.172	7.156	0.458	6.173	<0.001	50.157	8.010	0.523	6.262	<0.001	15.830	5.505	0.165	2.875	0.005
CSVD score											−3.884	0.309	−0.738	−12.578	<0.001
R^2^	0.514 (*F* = 61.23, *p* < 0.001)					0.520 (*F* = 49.21, *p* < 0.001)					0.829 (*F* = 144.82, *p* < 0.001)				
SCWT-C															
ORBinf.L	−108.803	20.840	−0.389	−5.221	<0.001	−103.492	25.290	−0.341	−4.092	<0.001	−33.908	11.825	−0.112	−2.867	0.005
IFGoperc.L	−97.777	16.293	−0.447	−6.001	<0.001	−110.365	18.495	−0.498	−5.967	<0.001	−21.534	9.464	−0.097	−2.275	0.025
CSVD score											10.056	0.531	0.826	18.935	<0.001
*R*^2^	0.507 (*F* = 60.17, *p* < 0.001)					0.516 (*F* = 48.94, *p* < 0.001)					0.904 (*F* = 284.71, *p* < 0.001)				

### 3.7 Mediation analysis

**Figure 3:**
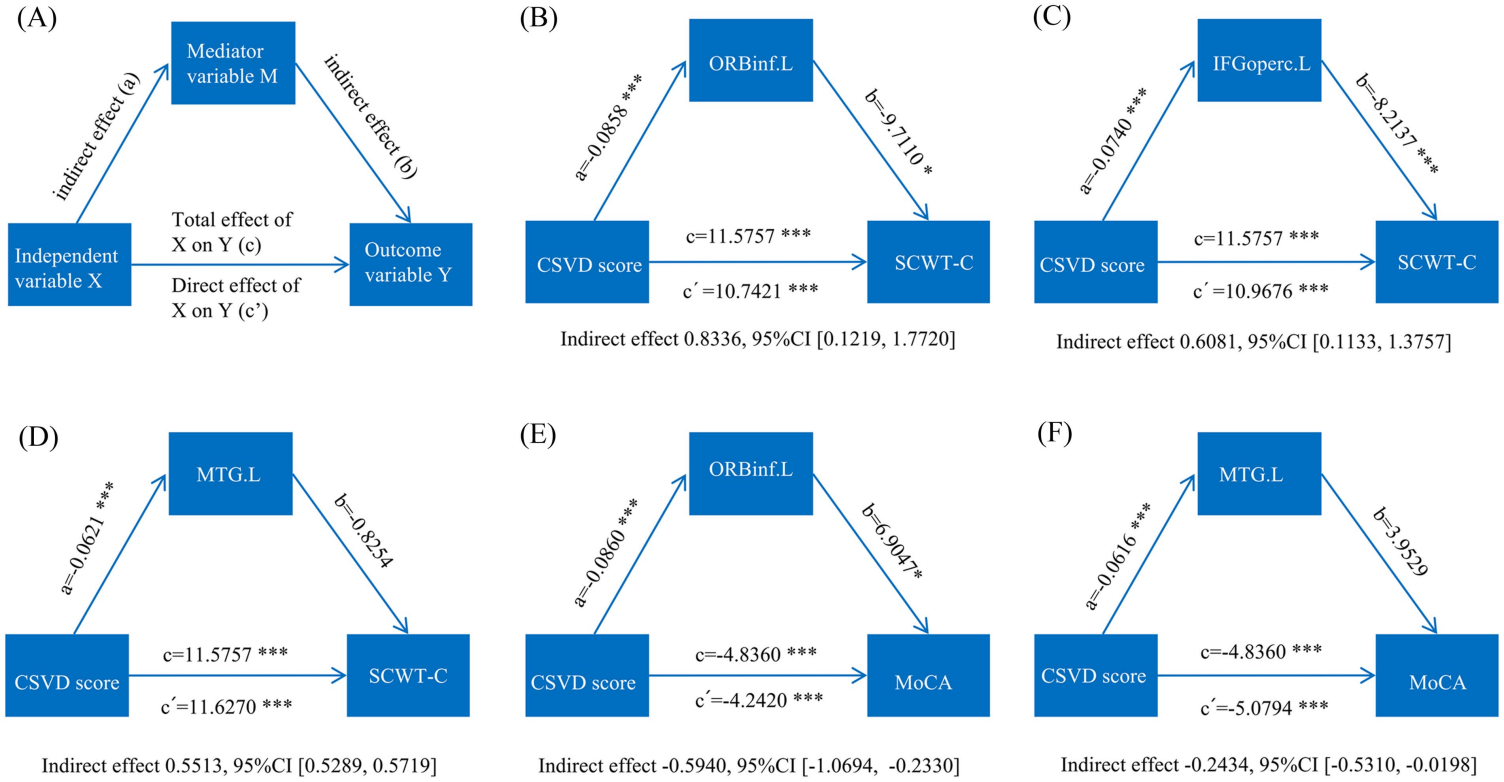
Schematic diagram illustrating mediation analyses of CSVD scores, CBF, and cognitive performance. **(A)** Schematic representation of mediation analysis: the total effect of the independent variable (X) on the outcome variable (Y) **(C)** is the sum of the indirect effect of X on Y through the mediating variable (M) and the direct effect of X on Y (c’); (B) Mediation analysis of CSVD scores (X) and SCWT-C scores (Y), with the CBF of ORBinf.L serving as the mediating variable (M); (C) Mediation analysis of CSVD scores (X) and SCWT-C scores (Y), with the CBF of IFGoperc.L as the mediating variable (M); **(D)** Mediation analysis of CSVD scores (X) and SCWT-C scores (Y), with the CBF of MTG.L as the mediating variable (M); **(E)** Mediation analysis of CSVD scores (X) and MoCA scores (Y), with the CBF of ORBinf.L as the mediating variable (M); **(F)** Mediation analysis of CSVD scores (X) and MoCA scores (Y) with the CBF of the MTG.L as the mediating variable (M). CI: confidence interval. ^*^p < 0.05.

MLRA demonstrated that the cognitive performance of the MetS-CSVD group correlated with CBF and CSVD scores. To further elucidate the relationship among CBF, CSVD scores, and cognitive performance (SCWT-C and MoCA), we used mediation analysis to set age, sex, and education as covariates. A schematic diagram of the mediation analysis was conducted (Figure 3A). The results exhibited that CBF significantly mediated the relationship between CSVD scores and cognitive performance, including the SCWT-C and MoCA scores (Figures 3B–F).

**Figure 4:**
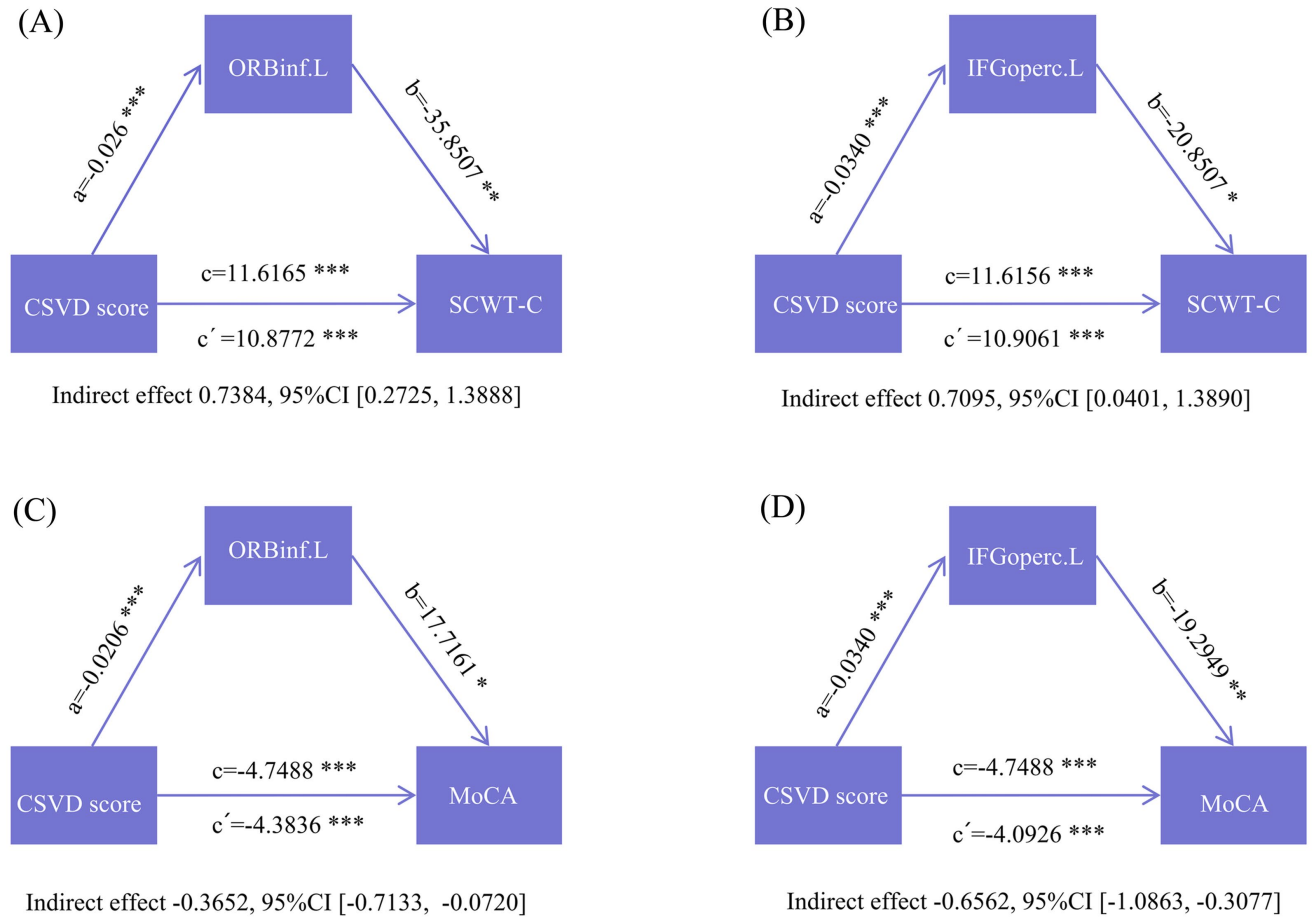
Schematic diagram of mediation analyses of CSVD scores, GMV, and cognitive performance. **(A)** Mediation analysis of the CSVD score (X) and SCWT-C (Y), with GMV of ORBinf.L as the mediating variable (M); **(B)** Mediation analysis of the CSVD score (X) and SCWT-C (Y), with GMV of IFGoperc.L as the mediating variable (M); **(C)** Mediation analysis of the CSVD score (X) and MoCA (Y); **(D)** Mediation analysis of the CSVD score (X) and MoCA, with GMV of ORBinf.L as the mediating variable (M).

Cognitive performance in patients with MetS-CSVD was also correlated with GMV and CSVD scores. Similarly, we applied mediation analysis to further clarify the relationship between CSVD scores, GMV, and cognitive performance using age, sex, and education as covariates. The results confirmed that GMV significantly mediated the relationship between CSVD scores and cognitive performance, as confirmed by SCWT-C and MoCA scores (Figures 4A–D).

## 4 Discussion

The mechanisms by which MetS causes cognitive impairment remain poorly understood. Our study was designed to investigate the relationships among CBF, GMV, and cognitive performance in patients with MetS-CSVD while providing structural imaging evidence for its pathophysiologic mechanisms. The results showed significantly lower overall cognitive scores, information processing speed, executive ability, attention, memory function, language, and visuospatial function in the MetS-CSVD group compared to the MetS-NCSVD and HC groups. Most of the under-perfused regions in patients with MetS-CSVD similarly demonstrated lower GMV, including the MFG.R, ORBinf.L, IFGoperc.L, MTG.L, and DCG.L. These brain regions were significantly lower in terms of both CBF and GMV. CBF was significantly lower in the ACG.L brain regions. CBF and GMV in the frontotemporal lobe were significantly related to overall cognitive function and executive function in the MetS-CSVD group. MLRA revealed that the CSVD score was the only factor independently and negatively associated with cognitive performance. CBF and GMV mediated the effect of the CSVD score on overall cognitive and executive function.

In the physiological state, the cerebral microcirculatory system regulates cerebral blood flow, thereby ensuring energy supply to neurons (István et al., 2021). CBF can maintain appropriate oxygen and nutrient supply through its regulatory mechanism, so despite changes in mean arterial pressure or cerebral vascular resistance, cerebral vascular self-regulation can still maintain relatively stable CBF (Paulson et al., 1990; Van Lieshout et al., 2003). Decreased cerebral perfusion can result in cerebral oxidative metabolic dysfunction and neuronal damage, which may further cause brain dysfunction. Moreover, evidence suggests that cerebral microvascular dysfunction may be driven by high blood sugar, insulin resistance, hypertension, and obesity (van Sloten et al., 2020). One study found that CBF reduction in patients with hypertension was concentrated in the frontal, parietal, and temporal lobes (Hajjar et al., 2010). Patients with type 2 diabetes mellitus present with disturbed brain neuronal activity and altered CBF (Ryan et al., 2014). Localized cerebral perfusion is deficient in diabetic patients compared to non-diabetic patients, and cognitive decline is associated with reduced CBF (Dai et al., 2017). A recent study exploring the effect of hypertension on cerebral cortical alterations in patients with diabetes confirmed that those with comorbid hypertension experienced an accelerated reduction in gray matter thickness (Shi et al., 2019). Hypertension can impair the blood supply system of the cerebral cortex by altering cerebrovascular microcirculatory hemodynamics (Petrie et al., 2018), resulting in prolonged cortical ischemia and subsequent atrophy (Jouvent et al., 2010). Herrmann et al. found that obesity is associated with reduced GMV in ORBinf.L, left middle, and right inferior frontal gyrus (Herrmann et al., 2019). A similar phenomenon was observed in our study: most apparently under-perfused brain regions, such as the frontal, temporal, and cingulate, exhibited a reduction in GMV.

Cognitive impairment in CSVD patients is associated with reduced cerebral perfusion. Our findings are consistent with the results of multiple studies; a recent study found that MetS has been linked to accelerated cognitive decline and related dementias via CSVD. However, the underlying mechanism of this relationship is unknown (Gosalia et al., 2024). Patients with symptomatic CSVD generally have lower CBF compared to patients with asymptomatic CSVD, with reduced perfusion in regions such as the temporal and frontal lobes correlating with the degree of cognitive impairment (Sun et al., 2016). CSVD severity is negatively correlated with CBF, and endothelial dysfunction and impaired self-regulation are key mechanisms. Endothelial dysfunction plays an essential role in the pathogenesis of atherosclerosis by inducing vasoconstriction, promoting a procoagulant state, enhancing inflammation, and stimulating cellular proliferation (Poggesi et al., 2016). Vascular resistance in small cerebral arteries is enhanced due to atherosclerosis, which may lead to inadequate perfusion and CSVD, disrupting blood flow and reducing oxygen supply to brain tissue, ultimately resulting in cognitive decline and neurodegeneration (Kivipelto et al., 2001). Another mechanism involves disrupting the blood–brain barrier, potentially decreasing cerebral blood flow (Cucullo et al., 2011). CSVD affects the entire brain, and as the disease progresses, blood flow to multiple regions, including the whole brain, decreases, leading to severe damage progression. Our findings indicate that patients with MetS-CSVD exhibit similar reductions in CBF and GMV, with reductions in GMV observed in CBF-reduced brain regions, particularly in the frontotemporal lobe. A longitudinal study revealed that MetS-associated hypertension and insulin resistance reduce GMV and cognitive decline by impairing cerebral microcirculation (Wu and Chen, 2021). GMV is extensively decreased in brain regions closely related to memory and executive functions, such as the hippocampus, thalamus, and parahippocampus in patients with CSVD-associated cognitive impairment (Mao et al., 2024). Chronic under-perfusion leads to impaired cortical integrity, with reduced CBF in the deep perforating arteries supplying subcortical nuclei, cortical projection fibers and connecting fibers, and disrupting communications between and within subcortical and cortical regions. Insufficient CBF perfusion to the cerebral cortex leads to structural changes in the brain; neither gray matter atrophy nor brain tissue atrophy was present in patients with type 2 diabetic dementia compared to non-demented controls, suggesting that inadequate cortical CBF perfusion precedes structural changes (Bangen et al., 2018). Reduced CBF can trigger a cascade of events, ultimately resulting in gray matter atrophy. Disruption of frontal-subcortical circuits has been implied as a potential underlying mechanism for cognitive impairment in CSVD (O'Sullivan et al., 2001).

Cognitive impairment in CSVD may result from damage to the cortico-subcortical pathway, disrupting the complex networks responsible for executive function and information processing (Bressler and Menon, 2010). The frontal lobes are part of the salience network and play vital roles in cognitive processes; therefore, reduced GMV in these regions may be involved in cognitive dysfunction in patients with CSVD, impairing cognition by disrupting white matter network pathways (Kim et al., 2014). White matter lesions cause dementia through a progressive disconnection syndrome resulting from damage to cortico-subcortical and cortico-cortical connections (Lawrence et al., 2013). Damage to fiber tracts in white matter and disruption of structural and functional connectivity between brain regions affect corresponding GMV (Schaefer et al., 2014; Du et al., 2019). Greater impairment in the fiber tracts in white matter is associated with more pronounced GMV reduction, significantly impacting both overall cognitive function and specific cognitive domains. Thus, WMH promotes GMV reduction and cognitive deterioration, proposing a possible mechanism through which CSVD impacts cognitive function by inducing neurodegenerative changes. Therefore, we suggest that MetS leads to cognitive impairment and vascular dementia by causing cerebral microvascular damage and CSVD, which subsequently reduces CBF, leading to gray matter atrophy, disrupted cortical connectivity, and white matter lesions. Obviously, the cognitive impairment mechanism caused by CSVD is far beyond the CBF and GMV changes we studied. A previous study found that glymphatic system dysfunction is independently associated with cognitive impairment in patients with CSVD after adjusting for six common risk factors (age, education, diabetes, hypertension, alcohol abuse, and smoking) and markers of CSVD (WMH, CMBs, LI, and EPVS) (Tang et al., 2022).

In summary, we applied ASL and VBM techniques to explore the role of cerebral perfusion and brain structure alteration mechanisms in patients with MetS-CSVD and their cognitive impairment. The findings suggest that CBF can be improved by preventing MetS, which in turn reduces the occurrence of CSVD and further improves brain structure and cognitive function. Our study has some limitations. First, our analyses were restricted to the MetS group; future studies should explore the unique role of MetS in brain perfusion, structural, and cognitive deficits more precisely by comparing the MetS-CSVD group with the CSVD-NMetS (CSVD without metabolic syndrome) group. Second, our study was cross-sectional, and the analytical methods for CBF and GMV have limited innovation, mainly relying on conventional techniques, which limits further analysis. In future research, emerging neuroimaging techniques, such as tensor-based morphometry and functional near-infrared spectroscopy, should be integrated to explore the relationship between neuroimaging characteristics and cognitive function in patients with MetS-CSVD. In addition, Mets encompass multiple abnormalities, such as diabetes, hypertension, and dyslipidemia. However, in this study, we did not conduct an in-depth analysis of which specific abnormalities have a key impact on brain structure and cognitive function. In our future research, we plan to use subgroup or mediating effect analysis to further explore the mechanisms by which different metabolic abnormalities contribute to brain abnormalities and cognitive impairment. Furthermore, confounding factors such as lifestyle factors (e.g., diet, physical activity) and comorbidities (e.g., sleep disorders) may also influence the results. We would consider confounding factors comprehensively in future studies.

## 5 Conclusion

Our findings suggest that in patients with MetS-CSVD, cognitive impairment is associated with MetS-promoted CSVD, which leads to reduced CBF, gray matter atrophy, disrupted cortical connectivity, and white matter lesions. These results provide new insights into the pathomechanisms of cognitive deterioration in patients with MetS-CSVD and suggest that regulating cerebral blood flow perfusion could be a potential strategy for preventing and treating cognitive impairment in patients with MetS-CSVD.

## Data Availability

The original contributions presented in the study are included in the article/Supplementary material, further inquiries can be directed to the corresponding author.

## Footnotes

^1^ http://www.fil.ion.ucl.ac.uk/spm

^2^ http://www.processmacro.org/

## Supplementary material

The Supplementary material for this article can befound online at: https://www.frontiersin.org/articles/10.3389/fnagi.2025.1538850/full#supplementary-material
