# Supplementary material for: Cerebral perfusion and gray matter volume alterations associated with cognitive impairment in metabolic syndrome with cerebral small vessel disease
Source: Front Aging Neurosci. 2025 Apr 22;17:1538850. doi: 10.3389/fnagi.2025.1538850 (PMC12053285; doi:10.3389/fnagi.2025.1538850)
Supplement: Supplementary file 1 [file Data_Sheet_1.pdf]

Supplementary Material

Cerebral Perfusion and Gray Matter Volume Alterations Associated with Cognitive Impairment in Metabolic Syndrome with Cerebral Small Vessel Disease

Han Liu[1,2,#],Wenhui Zheng[3,#], Jie Geng[1,4], Xia Zhou[1], Yu Xia[5], Yan Liu[6], Gang Zhang[1,7] , Xiaoqun Zhu[1], Daping Lv[1,2], Zhongwu Sun[1*]

[1] Department of Neurology, The First Affiliated Hospital of Anhui Medical University, Hefei, China

[2] Department of Neurology, Anhui Public Health Clinical Center, Hefei, China

[3] Department of Neurology, Tongren Hospital, Shanghai JiaoTong University School of Medicine, Shanghai, China

[4] Department of General Medicine, The Second Affiliated Hospital of Bengbu Medical University, Bengbu, China

[5] Department of Neurology, The Third People's Hospital of Hefei, Hefei, China

[6] Department of Neurology, Suzhou Hospital of Anhui Medical University, Suzhou, China

[7] Department of Rehabilitation, Anhui Public Health Clinical Center, Hefei, China.

[*] **Correspondence**: sunzhwu1964@163.com (S.Z.)

[#] These authors have contributed equally to this work.

1. Supplementary Materials and methods

Mediation analysis

This analysis assessed the overall effect of variable X on Y (c), which includes the indirect effect of X on Y through mediator M (a × b) and the direct effect of X on Y (c'). Significance tests employed 5000 bootstrap samples, with a significant indirect effect defined as a 95% confidence

interval (CI) that excluded zero. In addition, age, sex, education, and MetS factors were used as covariates. Statistical significance was set at $p < 0.05$.

2. Supplementary Tables and Figures

Supplementary Table 1. Correlation analysis between ROIs-CBF and neuropsychological test results in the MetS-CSVD group.

	MFG.R	ORBinf.L	IFGoperc.L	MTG.L	DCG.L	ACG.L
MoCA	0.021(0.824)	**0.534(<0.001)**	**0.499(<0.001)**	**0.408(<0.001)**	0.122(0.200)	0.166(0.568)
TMT-A	-0.171(0.083)	-0.060(0.621)	-0.065(0.897)	-0.059(0.886)	-0.078(0.429)	-0.075(0.720)
SCWT-A (dot)	-0.067(0.480)	-0.070(0.564)	-0.051(0.897)	-0.169(0.384)	-0.067(0.476)	-0.116(0.587)
SCWT-B (word)	-0.065(0.493)	-0.219(0.245)	-0.033(0.897)	-0.028(0.912)	-0.104(0.273)	-0.138(0.568)
TMT-B	-0.162(0.136)	-0.201(0.252)	-0.179(0.528)	-0.130(0.613)	-0.021(0.850)	-0.052(0.768)
SCWT-C (color word)	-0.024(0.802)	**-0.510(<0.001)**	**-0.494(<0.001)**	**-0.379(<0.001)**	-0.097(0.308)	-0.209(0.416)
DST (forward)	0.032(0.732)	0.020(0.835)	0.030(0.897)	0.031(0.942)	0.124(0.184)	0.040(0.768)
DST (backward)	0.107(0.252)	0.078(0.540)	0.093(0.648)	0.013(0.952)	0.015(0.877)	0.045(0.768)
AVLT-immediate recall	0.088(0.344)	0.079(0.540)	0.138(0.531)	0.020(0.952)	0.039(0.674)	0.100(0.651)
AVLT-delayed recall (5 min)	0.116(0.210)	0.083(0.540)	0.111(0.616)	0.006(0.952)	0.077(0.406)	0.125(0.576)
AVLT-delayed recall (20 min)	0.084(0.366)	0.111(0.464)	0.092(0.648)	0.085(0.736)	0.088(0.345)	0.144(0.568)
AVLT-recognition	0.049(0.598)	0.151(0.275)	0.011(0.946)	0.106(0.613)	0.021(0.820)	0.078(0.720)
VFT (animal)	0.059(0.533)	0.053(0.621)	0.026(0.897)	0.143(0.416)	0.090(0.341)	0.001(0.989)
VFT (fruit)	0.098(0.296)	0.102(0.501)	0.006(0.946)	0.072(0.800)	0.080(0.392)	0.011(0.964)
VFT (vegetable)	0.077(0.413)	0.157(0.275)	0.130(0.531)	0.156(0.412)	0.071(0.449)	0.083(0.720)
CDT	0.071(0.469)	0.148(0.299)	0.046(0.897)	0.040(0.942)	0.054(0.585)	0.065(0.749)

Note: Statistically significant differences are indicated using boldface type.

Abbreviations：MetS-CSVD：cerebral small vessel disease combined with metabolic syndrome；MoCA：Montreal cognitive assessment；TMT：Trail Making Test；SCWT：Stroop Color and Word Test；DST：Digit Span Test；AVLT：Auditory Verbal Learning Test；VFT：Verbal Fluency Test；CDT：Clock Drawing Test；MFG.R：Right middle frontal gyrus；ORBinf.L：Left inferior frontal orbital gyrus；IFGoperc.L：Left opercular part of inferior frontal gyrus； MTG.L：Left middle temporal gyrus；DCG.L：Left median cingulate and paracingulate gyri；ACG.L：Left anterior cingulate and paracingulate gyri.

Supplementary Table 2. Correlation analysis between ROIs-GMV and neuropsychological test results in MetS-CSVD group.

	MFG.R	ORBinf.L	IFGoperc.L	MTG.L	DCG.L
MoCA	0.120(0.195)	**0.553(<0.001)**	**0.490(<0.001)**	0.244(0.120)	0.033(0.727)
TMT-A	-0.019(0.852)	-0.067(0.785)	-0.093(0.603)	-0.039(0.794)	-0.094(0.343)
SCWT-A (dot)	-0.004(0.966)	-0.197(0.256)	-0.142(0.512)	-0.121(0.467)	-0.062(0.515)
SCWT-B (word)	-0.067(0.479)	-0.057(0.785)	-0.140(0.603)	-0.161(0.340)	-0.024(0.800)
TMT-B	-0.013(0.903)	-0.182(0.256)	-0.078(0.698)	-0.088(0.560)	-0.097(0.376)
SCWT-C (color word)	-0.023(0.811)	**-0.568(<0.001)**	**-0.463(<0.001)**	-0.228(0.120)	-0.068(0.476)
DST (forward)	0.022(0.814)	0.097(0.618)	0.039(0.742)	0.017(0.880)	0.007(0.940)
DST (backward)	0.086(0.357)	0.052(0.785)	0.045(0.742)	0.133(0.467)	0.050(0.595)
AVLT-immediate recall	0.030(0.745)	0.059(0.785)	-0.056(0.742)	0.077(0.560)	0.003(0.972)
AVLT-delayed recall (5 min)	0.082(0.379)	-0.043(0.805)	-0.084(0.603)	0.116(0.467)	0.040(0.665)
AVLT-delayed recall (20min)	0.128(0.167)	0.027(0.832)	-0.087(0.603)	0.104(0.467)	0.074(0.429)
AVLT-recognition	0.013(0.890)	0.035(0.816)	-0.037(0.742)	0.019(0.880)	0.038(0.681)
VFT (animal)	0.050(0.592)	0.158(0.256)	0.089(0.603)	0.087(0.560)	0.053(0.570)
VFT (fruit)	0.096(0.307)	0.169(0.256)	0.015(0.873)	0.190(0.240)	0.034(0.716)
VFT (vegetable)	0.085(0.366)	0.115(0.523)	0.157(0.506)	0.106(0.467)	0.023(0.809)
CDT	0.031(0.755)	0.021(0.838)	0.112(0.603)	0.045(0.794)	0.157(0.109)

Note: Statistically significant differences are indicated using boldface type.

Abbreviations：MetS-CSVD：cerebral small vessel disease combined with metabolic syndrome；MoCA：Montreal cognitive assessment；TMT：Trail Making Test；SCWT：Stroop Color and Word Test；DST：Digit Span Test；AVLT：Auditory Verbal Learning Test；VFT：Verbal Fluency Test；CDT：Clock Drawing Test；MFG.R：Right middle frontal gyrus；ORBinf.L：Left inferior frontal orbital gyrus；IFGoperc.L：Left opercular part of inferior frontal gyrus； MTG.L：Left middle temporal gyrus；DCG.L：Left median cingulate and paracingulate gyri；ACG.L：Left anterior cingulate and paracingulate gyri.

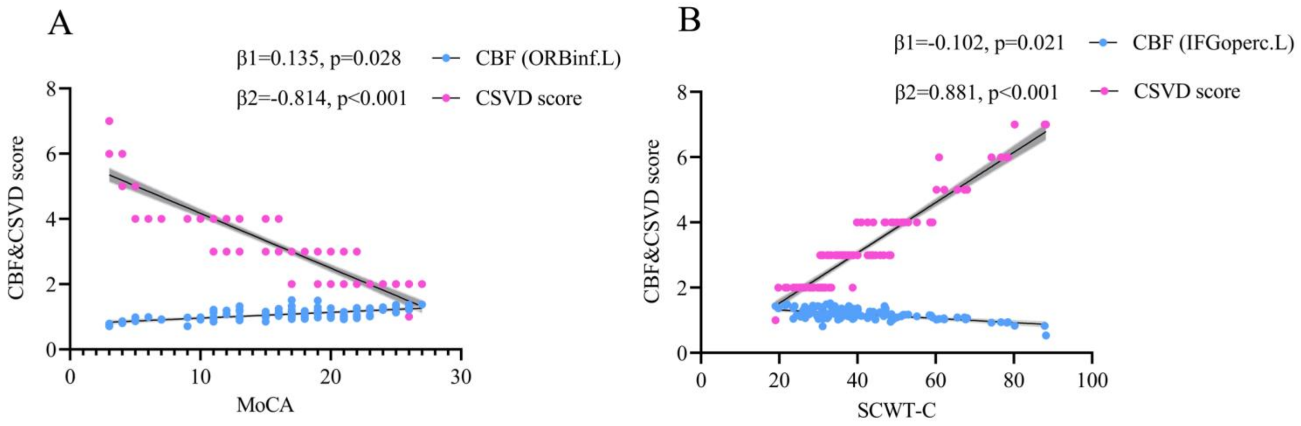

Supplementary Figure 1. Scatterplots of the relation between CBF, CSVD and cognition function. (A) Relation between CBF, CSVD and MoCA. (B) Relation between CBF, CSVD and SCWT-C.

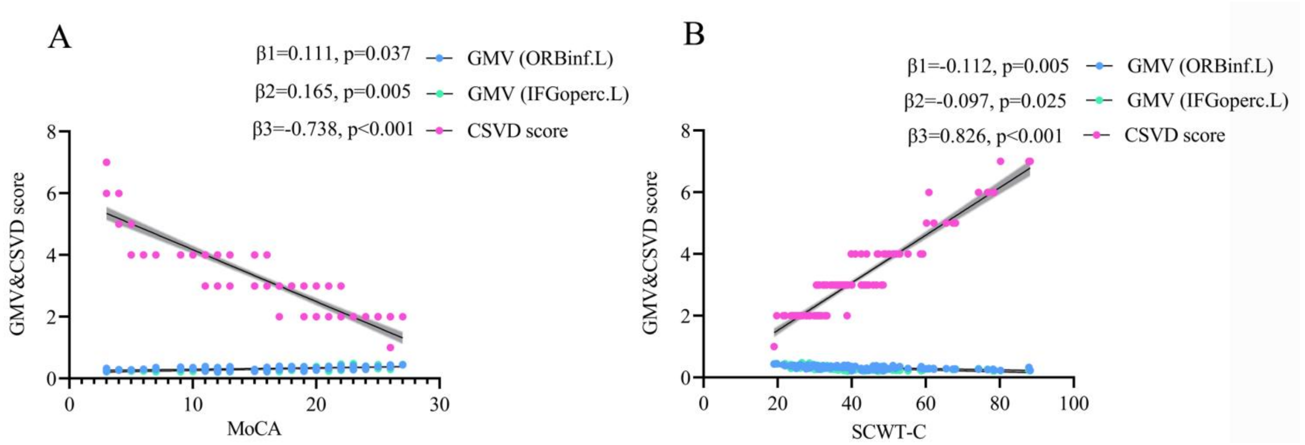

Supplementary Figure 2. Scatterplots of the relation between GMV, CSVD and cognition function. (A) Relation between GMV, CSVD and MoCA. (B) Relation between GMV, CSVD and SCWT-C.
